# A purified and lyophilized *Pseudomonas aeruginosa* derived pyocyanin induces promising apoptotic and necrotic activities against MCF-7 human breast adenocarcinoma

**DOI:** 10.1186/s12934-022-01988-x

**Published:** 2022-12-17

**Authors:** Ahmed A. Abdelaziz, Amal M. Abo Kamer, Khaled B. Al-Monofy, Lamiaa A. Al-Madboly

**Affiliations:** Department of Pharmaceutical Microbiology, Faculty of Pharmacy, Tanta University, Tanta, Egypt

**Keywords:** Bioprospection, Pyocyanin, Anticancer, Caspase-3, Human breast adenocarcinoma (MCF-7)

## Abstract

**Background:**

Pyocyanin, a specific extracellular secondary metabolite pigment produced by *Pseudomonas aeruginosa*, exhibits redox activity and has toxic effects on mammalian cells, making it a new and potent alternative for treating cancer. Breast cancer (BC) treatment is now defied by acquired and de novo resistance to chemotherapy, radiation, or targeted therapies. Therefore, the anticancer activity of purified and characterized pyocyanin was examined against BC in our study.

**Results:**

The maximum production of pyocyanin (53 µg/ml) was achieved by incubation of the highest pyocyanin-producing *P. aeruginosa* strain (P32) in pH-adjusted peptone water supplemented with 3% cetrimide under shaking conditions at 37 °C for 3 days. The high purity of the extracted pyocyanin was proven by HPLC against standard pyocyanin. The stability of pyocyanin was affected by the solvent in which it was stored. Therefore, the purified pyocyanin extract was lyophilized to increase its shelf-life up to one year. Using the MTT assay, we reported, for the first time, the cytotoxic effect of pyocyanin against human breast adenocarcinoma (MCF-7) with IC_50_ = 15 μg/ml while it recorded a safe concentration against human peripheral blood mononuclear cells (PBMCs). The anticancer potential of pyocyanin against MCF-7 was associated with its apoptotic and necrotic activities which were confirmed qualitatively and quantitively using confocal laser scanning microscopy, inverted microscopy, and flow cytometry. Caspase-3 measurements, using real-time PCR and western blot, revealed that pyocyanin exerted its apoptotic activity against MCF-7 through caspase-3 activation.

**Conclusion:**

Our work demonstrated that pyocyanin may be an ideal anticancer candidate, specific to cancer cells, for treating MCF-7 by its necrotic and caspase-3-dependent apoptotic activities.

## Background

Breast cancer (BC) is the most common type of cancer in women, there were almost two million new cases found in the past decade, with total BC representing 11.6% of all cancer cases according to statistics from around the world [1]. Chemotherapy drugs are the backbone of cancer treatment by targeting the tumor cells and producing reactive oxygen species which destroy tumor cells [2]. However, chemotherapy affects normal cells which leads to various dose-dependent side effects such as vomiting, fatigue, hair loss, nausea, and even death in life-threatening cases [3]. Resistance to chemotherapeutic interventions, because of extended treatment, remains a great challenge in clinical management for breast cancer patients [4]. Therefore, if cancer cannot be cured, new therapeutic advancements will be required for the treatment of cancer [5].

Pyocyanin, a water-soluble, blue-green, nitrogen-containing phenazine pigment, is produced by 90–95% of *Pseudomonas aeruginosa* isolates [6]. The pigment has several benefits, including: (1) being natural, biodegradable, and eco-friendly; (2) being easy to manipulate and selecting strains with the highest yield using straightforward cultivation techniques and inexpensive substrates; (3) being able to be used to produce biomass; and (4) having a quick and straightforward collection and extraction procedures in comparison to other chemical synthesis procedures [7]. Therefore, pyocyanin is considered a fantastic research pigment and may be used in the fields of science, medicine, nutrition, the environment, and energy generation [8].

Excessive Reactive oxygen species (ROS) led to caspase-3 activation which potentiates the cancer cell death process by apoptosis [9]. Pyocyanin, a low-molecular-weight (210 Da) zwitterion, easily diffuses through cell membranes and increases intracellular levels of ROS, especially superoxide and hydrogen peroxide, under aerobic conditions by cyclic non-enzymatic reduction by NAD(P)H [10–12]. The apoptotic activity of pyocyanin was observed against cancer cell lines such as rhabdomyosarcoma (RD), hepatocellular carcinoma (HepG2), and human pancreatic cancer (PANC-1) [11, 13, 14]. As well as the cell lines exposed to a high concentration of pyocyanin showed a necrotic morphological change [15].

Consequently, the goals of the current study include the optimization of extrinsic factors affecting pyocyanin output, purification, characterization of pyocyanin, and determination of the cytotoxicity of produced pyocyanin against normal human cells as well as testing the anticancer activity of the purified and characterized pyocyanin, for the first time, against human breast adenocarcinoma (MCF-7).

## Results

### Bacterial strains

In the present study, a total of 100 isolates from different sources (sputum (48), urine (32), wound / pus (14), and blood (6)) were identified as *P. aeruginosa* based on their morphological and biochemical characteristics and were investigated for pyocyanin production.

### Pyocyanin determination

**Fig. 1:**
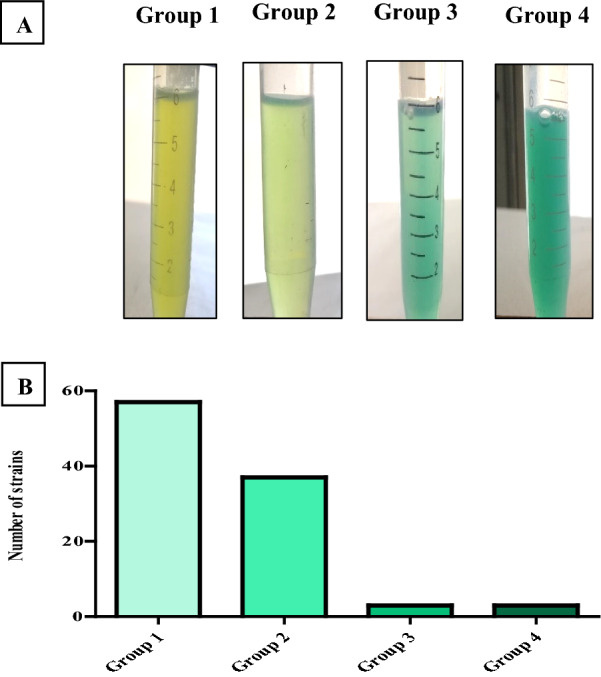
Categorization of *P. aeruginosa* strains into four groups according to the amount of pyocyanin produced by each strain. **A** Representative *P. aeruginosa* strain from each group. **B** The number of *P. aeruginosa* strains in each group

All *P. aeruginosa* strains were tested for pyocyanin pigment production, but the amount of pyocyanin produced by each *P. aeruginosa* strain was different. *P. aeruginosa* strains were divided into four groups based on the concentration of produced pyocyanin as shown in (Fig. 1). In group 1 (57%) the clinical strains produced pyocyanin 5 µg/ml or less. In group 2 (37%) the clinical strains produced pyocyanin 5 µg/ml to a maximum of 10 µg/ml. In group 3 (3%) the clinical strains produced pyocyanin of more than 10 µg/ml. In group 4 (3%) the clinical isolates that produced pyocyanin of more than 15 µg/ml were recorded for strains P 32, P 201, and P 258, which were then selected for the next step.

### Optimization of pyocyanin production conditions

**Fig. 2:**
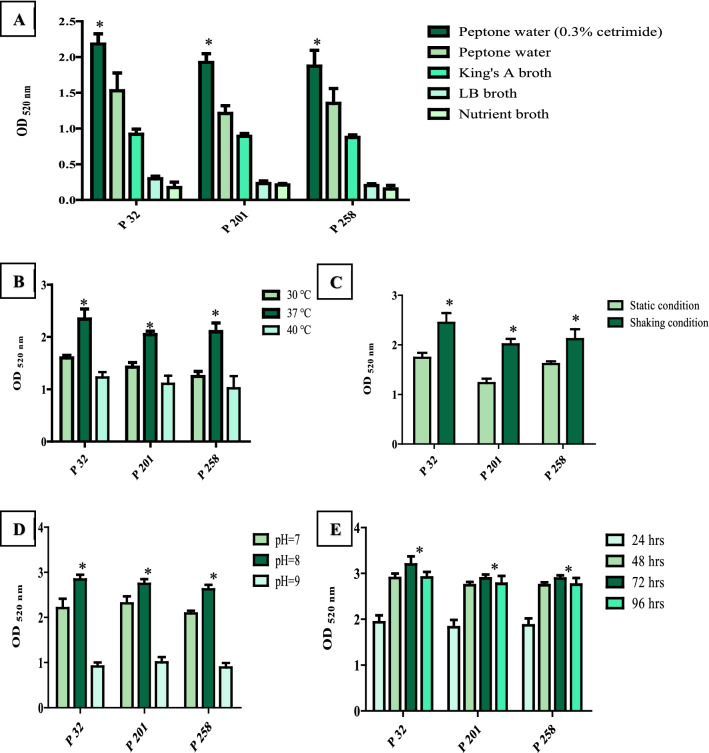
Effect of the different conditions on pyocyanin production by P 32, P 201, and P 258. **A** Effect of incubation media. **B** Effect of incubation temperature. **C** Effect of shaking and static conditions. **D** Effect of pH. **E** Effect of incubation time. The error bars indicate standard deviations. The asterisks represent statistical significance (*P* < 0.05)

There was a substantial difference in pyocyanin production between the examined culture media. the order of examined media according to pyocyanin yield was peptone water supplemented with 0.3% cetrimide > peptone water > king’s A broth > LB broth > nutrient broth, and the most productive *P. aeruginosa* strain was P 32 (OD_520 nm_ = 2.17) as shown in (Fig. 2A). Pyocyanin yield at 37 °C by the three tested strains was greater than pyocyanin yield at 30 °C and 40 °C while the most productive strain was P 32 (OD _520 nm_ = 2.34) as shown in (Fig. 2B). Shaking inoculated flasks caused a significant increase (*P* < 0.05) in pyocyanin yield compared to static conditions and the most productive strain under shaking incubation was P 32 (OD _520 nm_ = 2.643) as shown in (Fig. 2C). There was a significant increase (*P* < 0.05) in pyocyanin yield when P 32, P 201, and P 258 strains were grown at pH = 8 in comparison to pyocyanin yield at pH = 7 and pH = 9, and P 32 was the most productive strain at pH = 8 (OD _520 nm_ = 2.843) as shown in (Fig. 2D). Incubation time ranged from 24 to 96 h led to a maximum pyocyanin yield after 3 days and the most productive strain was P 32 (OD _520 nm_ = 3.2) as shown in (Fig. 2E). Therefore, obtaining the maximum pyocyanin yield was achieved by incubation *P. aeruginosa* in a previously pH-adjusted (pH = 8) peptone water medium supplemented with 0.3% cetrimide under shaking conditions at 37 °C for three days and the preferred *P. aeruginosa* strain was P 32.

### Mass production and extraction of pyocyanin

**Fig. 3:**
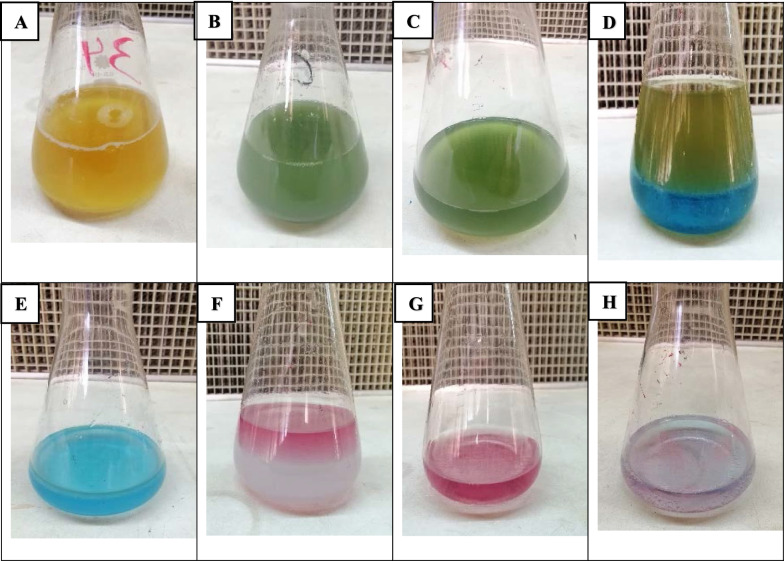
Extraction of pyocyanin pigment produced by *P. aeruginosa* (P 32). **A** Inoculation of P 32 in peptone medium supplemented with 0.3% cetrimide in a 250 ml Erlenmeyer flask; **B** Pyocyanin production after 3 days of incubations; **C** Collection of supernatant after centrifugation; **D** Addition of chloroform layer; **E** Separation of chloroform layer; **F** Addition of 0.2 N HCl; **G** Separation of the aqueous layer; **H** Addition of 0.2 N NaOH and restore the blue color

**Fig. 4:**
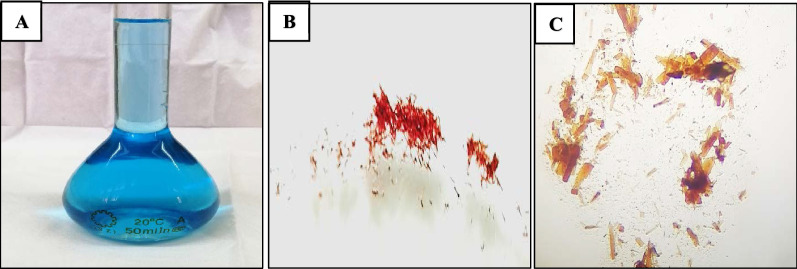
**A** Silica gel purified pyocyanin. **B** Lyophilized pyocyanin powder. **C** lyophilized pyocyanin powder was examined at 100 × magnification (LABOMED, CXL, USA)

Blue-green pigment pyocyanin was produced during the growth of *P. aeruginosa* in a 250 ml Erlenmeyer flask containing previously pH-adjusted (pH = 8) peptone water medium supplemented with 0.3% cetrimide and incubated at 37 °C for 3 days. After the centrifugation process and collecting the supernatant, pyocyanin was extracted using chloroform/0.2 N HCl as shown in (Fig. 3). The extraction of pyocyanin was based on its redox properties and on the fact that only pure pyocyanin when it passes from basic to acidic pH, changes its color. This dualism in solubility makes the purification of pyocyanin promising and highly effective by altering the pH and water/chloroform extraction Fig. 4.

### Purification and lyophilization of pyocyanin

Like most low-molecular weight compounds, the crude pyocyanin of the selected *P. aeruginosa* strain (P 32) was purified using column chromatography. When pyocyanin was eluted with 15% methanol in chloroform, crude pigment fractions on the silica gel column appeared in yellow-green, light blue, and dark blue bands. The blue fractions were collected and analyzed by UV–Vis spectrophotometer in comparison to standard pyocyanin, whereas the blue band was easily eluted as pure pyocyanin. As a reason for the low stability of pyocyanin, the extracted pyocyanin was lyophilized to increase its stability and stored at − 18 °C for further studies. The solubility of the lyophilized pyocyanin was investigated; results revealed that the pigment easily dissolved in chloroform, HCl, and water.

### Characterization of lyophilized pyocyanin

**Fig. 5:**
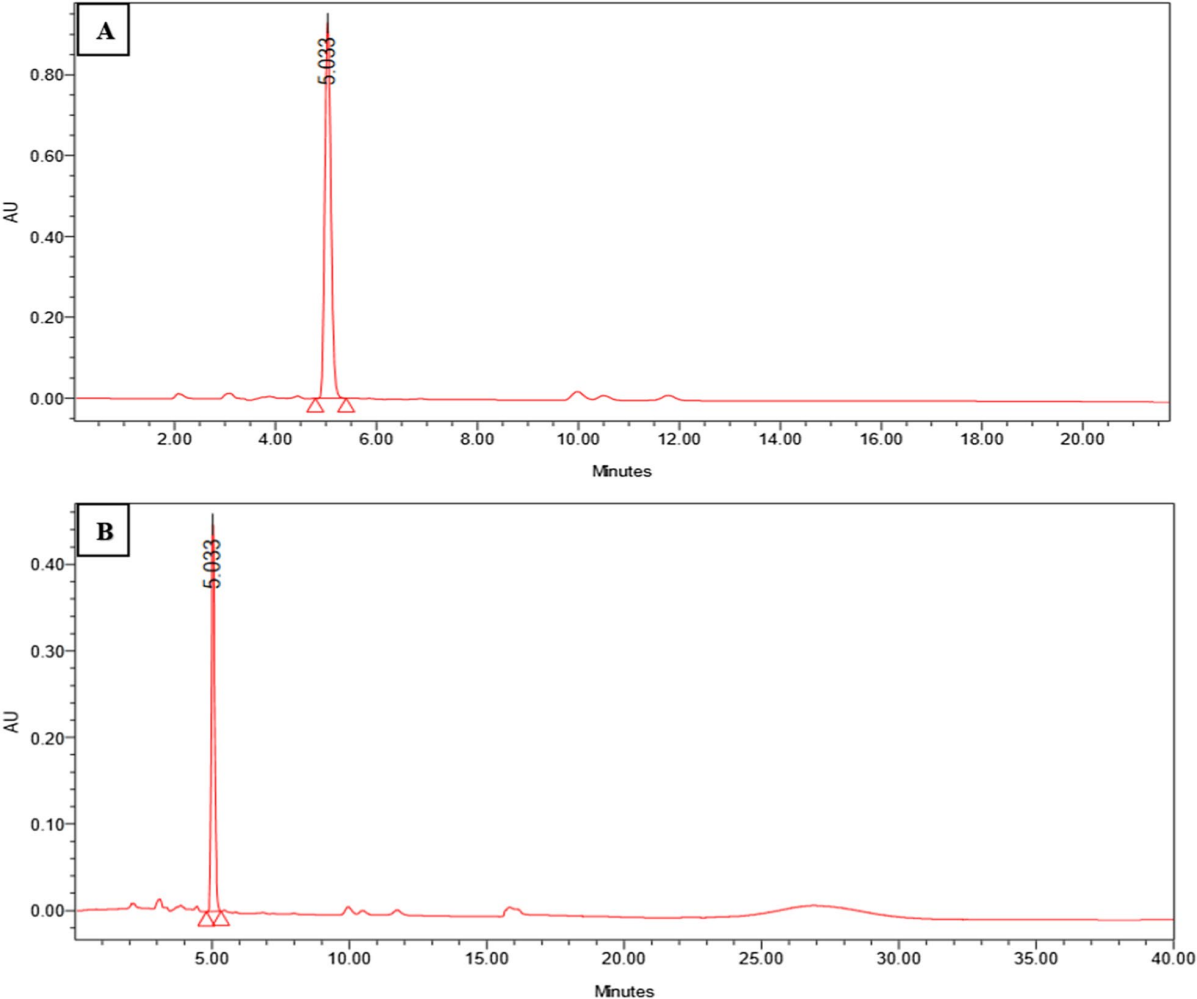
Chromatograms of pyocyanin. **A** Standard pyocyanin. **B** Extracted pyocyanin using HPLC showing the same retention time at 5.033 min

The high-performance liquid chromatography system (HPLC system) was employed to analyze the lyophilized pyocyanin and compare it with a commercial standard (Sigma-Aldrich, St Louis, MO, USA). The HPLC chromatogram showed a single peak with a retention time of 5.033 min for standard pyocyanin, which was comparable to that of the extracted lyophilized pyocyanin, which suggested that it was the same molecule as shown in (Fig. 5).

### Stability of lyophilized pyocyanin

**Fig. 6:**
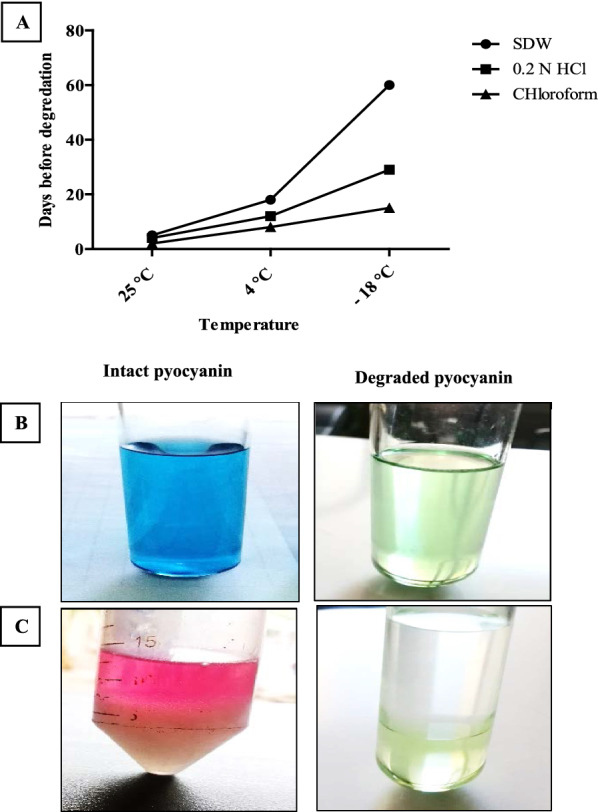
**A** Pyocyanin degradation over days in different solvents at different temperatures. **B** Pyocyanin lost its blue color due to degradation over days. **C** Loss of dual solubility of pyocyanin prevented its transfer to aqueous phase after adding 0.2 N HCl to chloroform containing pyocyanin.

**Fig. 7:**
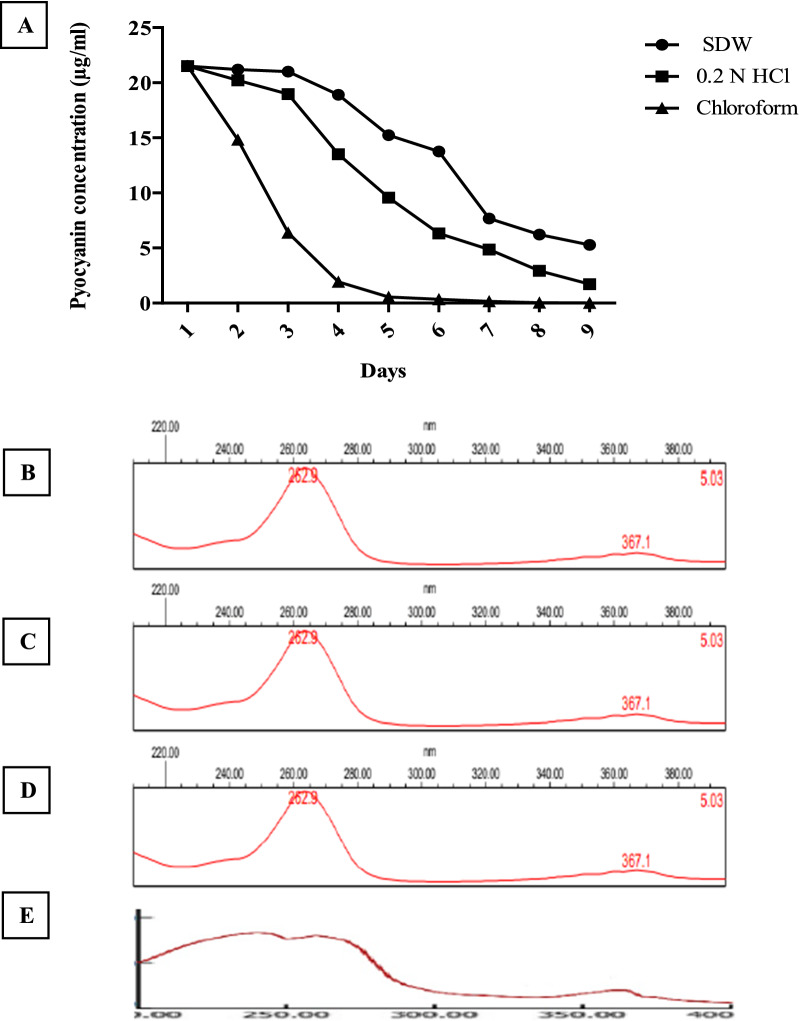
**A** Reduction in pyocyanin concentration stored in different solvents at 4 °C over days. UV–Vis spectrums of **B** standard pyocyanin, **C** extracted pyocyanin, **D** one-year stored pyocyanin at – 18 °C, and **E** extracted pyocyanin after color change (degraded pyocyanin)

**Fig. 8:**
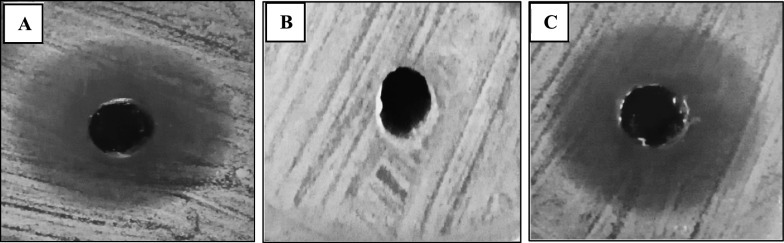
Inhibition zones produced by pyocyanin against *Escherichia coli*. **A** Freshly prepared pyocyanin in SDW. **B** 60 days stored pyocyanin in SDW at – 18 °C. **C** Freshly dissolved pyocyanin in SDW after 1 year of storage at − 18 °C

The stability studies were performed to determine the most favorable conditions for storing our produced pyocyanin powder for further application. We dissolved the same amount of pyocyanin in different solvents at different temperatures and observed the known characteristics of pure pyocyanin such as its colors and dualism capacity. The pyocyanin concentration over days and its characteristic UV–VIS spectrum were also observed. Pyocyanin color disappeared over days as well as its ability to transfer from the organic phase to the aqueous phase was lost upon degradation as shown in (Fig. 6). The concentration of pyocyanin was decreased by days due to its degradation; the degradation rate was faster in chloroform and 0.2 N HCl compared to sterile distilled water as shown in figure (Fig. 7A). The extracted pyocyanin showed two maximum peaks of 262.9 and 367.1 nm, like the standard commercial product. Pyocyanin degradation over days makes changes in the UV–Vis spectrum showing 3 peaks, instead of 2 peaks, at 240, 260, and 365 nm as shown in figure (Fig. 7B–E). From the above data, the preferred solvent and temperature were SDW at − 18 °C. For further estimation of stability over long period storage of dissolved and lyophilized pyocyanin, we checked its characteristic antibacterial activity for freshly prepared pyocyanin in SDW, 60 days stored pyocyanin in SDW, and one-year stored lyophilized form at − 18 °C. Stored lyophilized pyocyanin powder conserved its antibacterial activity producing the same inhibition zones which produced by freshly prepared pyocyanin in SDW while stored pyocyanin solution lost its antibacterial activity as shown in (Fig. 8). Therefore, storing lyophilized pyocyanin powder at − 18 °C is the optimal approach for pyocyanin conservation for further application.

### Evaluation of pyocyanin safe concentrations using human peripheral blood mononuclear cells (PBMCs)

**Fig. 9:**
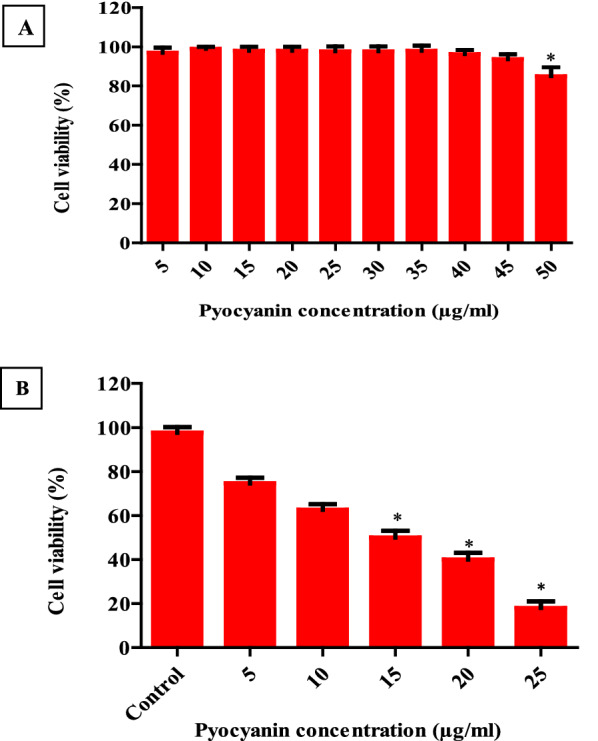
The cytotoxic effect of pyocyanin on normal human cell PBMCs (**A**) and on MCF-7 cells using MTT assay **B** showed selective cytotoxicity on MCF-7 at concentrations below 45 μg/ml without affecting normal human cells. The error bars indicate standard deviations. The asterisks represent statistical significance (*P* < 0.05).

For determination the sub-toxic concentrations of pyocyanin on normal human cells which will be used later for its useful applications, normal human PBMCs were incubated for 48 h with increasing pyocyanin concentrations, and then the cytotoxic activity was measured by the MTT assay. The viability percentage of PBMCs remained constant (nearly 100%) until reaching a concentration of 45 µg/ml as shown in (Fig. 9A). These results indicated that pyocyanin was tolerated by normal human cells and pyocyanin can be used as a safe agent in human at a concentration below 45 µg/ml.

### In Vitro cytotoxic effect of pyocyanin

The MTT assay was used to evaluate the in vitro cytotoxic activity of pyocyanin extract on the MCF-7 cell line at concentrations below 45 µg/ml. MTT assay was introduced for the estimation of cell viability by Mosmann because he demonstrated that MTT reduction to formazan is proportional to the number of metabolically viable cells in the culture. Pyocyanin led to a significant (*P* < 0.05) dose-dependent reduction in the cell viability of MCF-7 as shown in (Fig. 9B). The concentration of pyocyanin which led to 50% of the viable population was 15 μg/ml (IC_50_).

### Necrosis and apoptosis analysis using confocal laser scanning microscopy (CLSM)

**Fig. 10:**
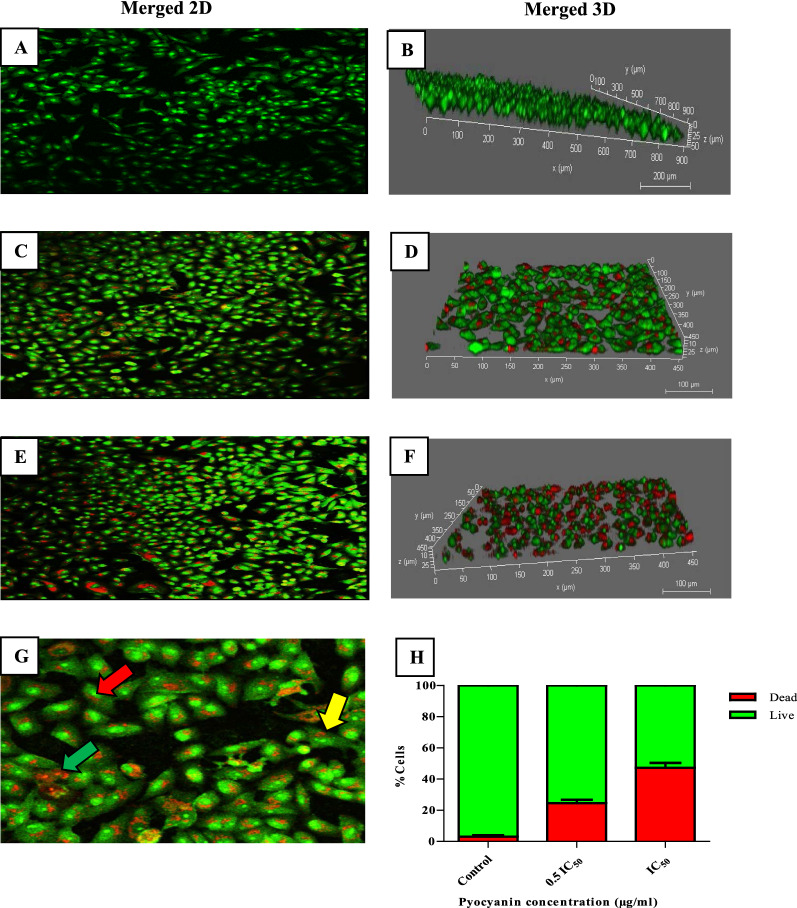
Fluorescent micrographs (10 × magnification) of double-stained MCF-7 cells by propidium iodide and acridine orange (PI/AO) stains after 24 h of pyocyanin treatment. **A**, **B** Viable untreated MCF-7 cells emit green fluorescence. Red fluorescence emission indicates the formation of apoptotic and necrotic cells after 24 h treatment of MCF-7 with 0.5 IC_50_ (**C**, **D)** and IC_50_ (**E**, **F**) of pyocyanin. **G** Fluorescent micrographs (20 × magnification) of MCF-7 cells showing membrane blebbing (yellow arrow), late apoptotic cells (red arrow), and necrotic cells (green arrow). **H** Quantitative data representing dead and viable cell before and after pyocyanin treatment with 0.5 IC_50_ and IC_50_

Acridine orange and propidium iodide AO/PI double stained untreated and pyocyanin-treated MCF‐7 cells with 0.5 IC_50_ and IC_50_ were examined using CLSM for detection the induction of apoptosis and necrosis followed by pyocyanin treatment. As shown in (Fig. 10), untreated MCF‐7 cells were viable and emitted green fluorescence light with clear, complete, and defined shapes. In contrast, pyocyanin-treated MCF‐7 cells emitted red fluorescence light which occurred due to the formation of apoptotic and necrotic. The treated MCF-7 cells exhibited the morphological changes of apoptotic cells such as cell shrinkage and membrane blebbing which led to a reduction of the thickness of monolayer cells after treatment by 50% as shown in (Fig. 10) (merged 3D).

### Apoptosis and necrosis analysis using flow cytometry

**Fig. 11:**
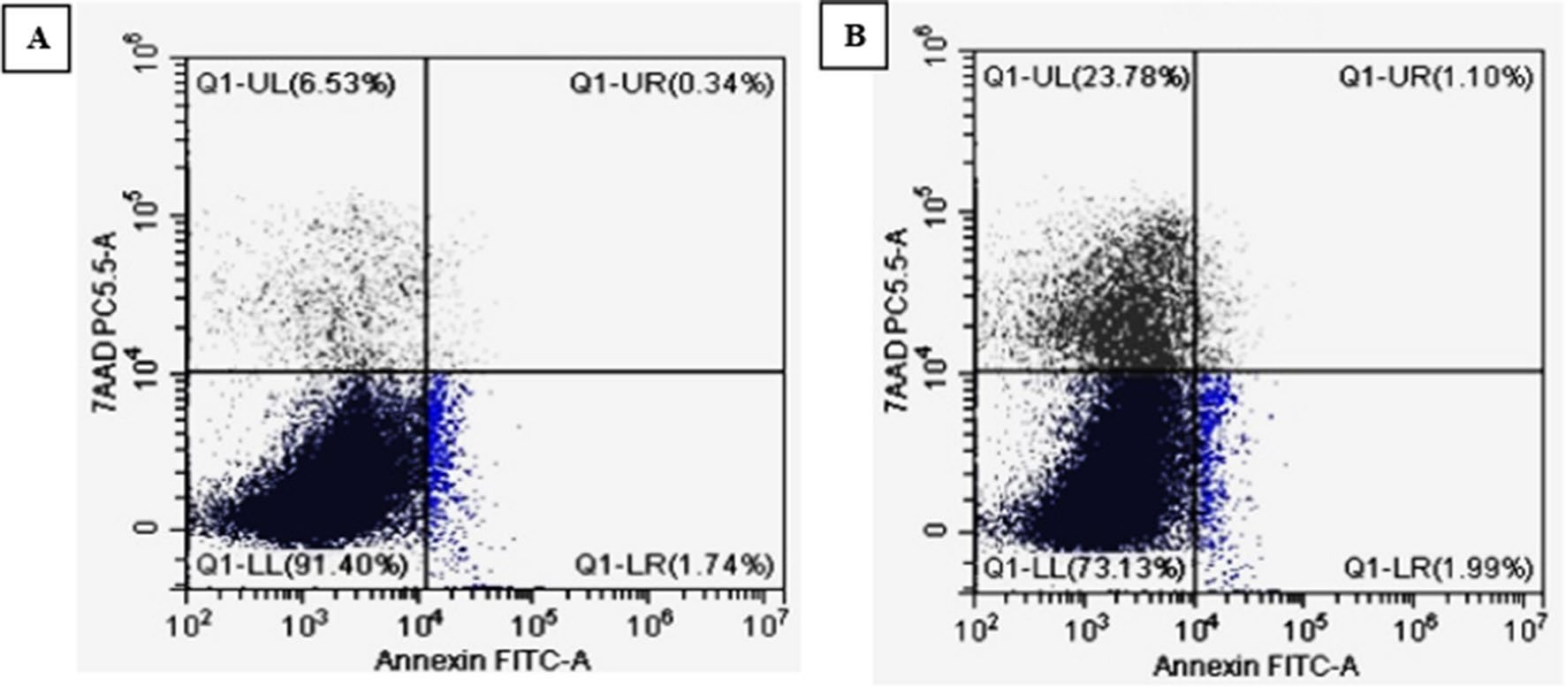
Illustrative dot plots obtained from flow cytometry analysis of untreated MCF-7 cells (**A**) and pyocyanin-treated MCF-7 cells (IC_50_) (**B**) after staining with Annexin V-FITC/PI stains showing induction of apoptosis and necrosis after 24 h of treatment. Q1-LL indicates live cells, Q1-LR indicates early apoptotic cells, Q1-UL indicates necrotic cells, and Q1-UR indicates late apoptotic cells

To further prove whether the inhibition of MCF-7 cell growth after pyocyanin treatment was associated with the induction of apoptosis and necrosis or not. The untreated and treated MCF-7 cells were stained by Annexin V-FITC and PI stains and analyzed by Novocyte Flow Cytometer. As revealed in (Fig. 11), the early apoptotic cells increased from 1.74 to 1.99%, and the late apoptotic cells increased from 0.34 to 1.1%, more than 3 folds, while the necrotic cells increased from 6.53 to 23.78%, more than 3 folds, after treating the cells with pyocyanin at IC_50._ The above data confirm that the anticancer activity of pyocyanin against MCF-7 cells includes activation of apoptosis and necrosis.

## Morphological changes detection using inverted microscopy

**Fig. 12:**
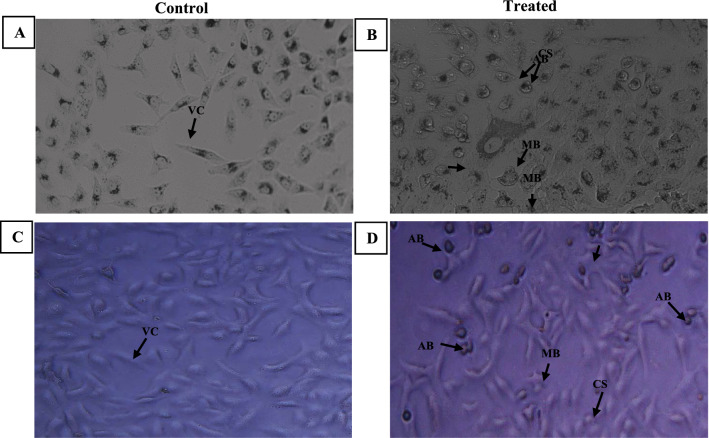
Morphological changes in MCF-7 after 24 h treatment with pyocyanin (IC_50_) observed by inverted microscope at magnification of 40 x. **A**, **C** The untreated MCF‐7 cells displayed normal cell shape with a clear and typical cell membrane **VC**. **B**, **D** The treated MCF‐7 cells experienced cell shrinkage CS, blebbing of membrane MB, and formation of apoptotic bodies AB

Morphological changes in MCF-7 cells occurred because of the induction of apoptosis and necrosis after pyocyanin treatment. Cells of MCF-7, after pyocyanin treatment, became shrunk in size, round, and disconnected from the monolayer surface of the wells. The number of MCF-7 cells was also lessened in comparison with untreated MCF-7 cells as well as some of the treated cells revealed membrane blebbing, a hallmark of apoptosis, and the formation of apoptotic bodies which appeared oval or round masses smaller than the original cells as shown in (Fig. 12).

### Caspase-3 measurement using real-time pcr and western blot

**Fig. 13:**
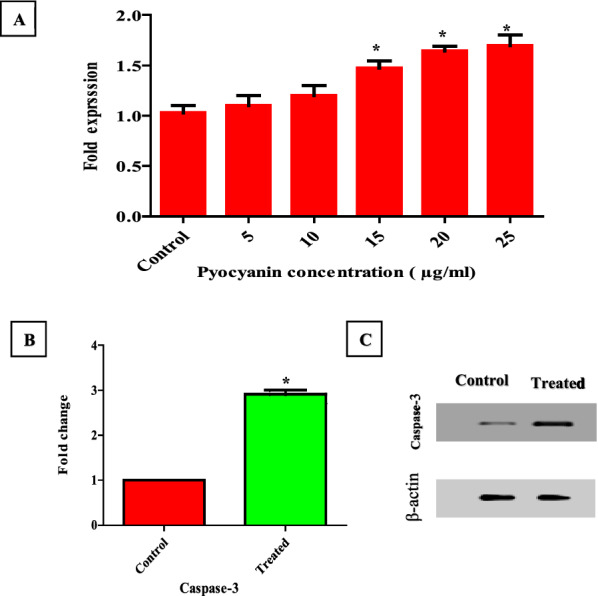
Caspase-3 dependent apoptotic activity of pyocyanin in MCF-7 cells. **A** mRNA expression of caspase-3 was determined using real-time PCR assays. **B, C** The protein level of caspase 3 in MCF-7 cells treated with pyocyanin (IC_50_) was determined using Western blot assays, and β-Actin was used as an internal control. The error bars indicate standard deviations. The asterisks represent statistical significance (*P* < 0.05)

Caspase-3 is a potential therapeutic target for anticancer agents and plays a pivotal role in apoptosis. Selective caspase-3 inhibition, on the other hand, is associated with cell death inhibition. Real-time PCR was performed using caspase-3 specific primers in pyocyanin-treated MCF-7 cells. Caspase-3 was significantly (*P* < 0.05) overexpressed in treated MCF-7 cells compared to untreated cells as shown in (Fig. 13A). Finally, the expression level of apoptosis-related active protein caspase-3 was confirmed by Western blot assay. The expression of active caspase-3 protein was clearly elevated by pyocyanin treatment (IC_50_) compared to control group as shown in (Fig. 13B, C). Our results revealed that pyocyanin-induced apoptosis in the human breast Adenocarcinoma cell line (MCF-7) was dependent on caspase-3 activation.

## Discussion

Cancer is one of the most leading causes of mortality worldwide and the prevence of this disease is increasing more quickly [16]. Chemotherapy is the cornerstone for treatment of many cancers at various stages of the disease. Resistance to chemotherapy, resulting from a variety of tumor or host-related causes, leads to therapeutic failure and ultimately death [17]. Furthermore, chemotherapy can have negative side effects on healthy cells and tissues, including alopecia, nausea, vomiting, and a reduction in bone marrow function [18]. Hence, the ongoing search for anticancer agents, as an alternative to chemotherapy, has been the subject of numerous investigations [19–21].

The biodegradability and safety of natural pigments compared to synthetic pigments attracts the attention of the industry for human use [22]. Despite the diversity of natural pigments, microbial pigments are favored due to simple and rapid pigment extraction and scaling up. The safety of bacterial pigments, such as violacein, indigoidine, melanin, carotenoids, prodigiosin, rhodopsins, pyoverdine, and pyocyanin, provokes their applications in the pharmaceutical, cosmetics, food industries, and textiles [22–24]. The therapeutic personality of bacterial pigments is related to their antioxidant, antimicrobial, cytotoxic, and anticancer properties [23]. In our study, we focused on the anticancer activity of pyocyanin against BC.

In our study, pyocyanin production estimates revealed that all *P. aeruginosa* strains could manufacture the pigment in varying amounts (0.14 to 21.5 µg/ml). The variation in pyocyanin production across the studied strains was explained by the presence or absence of specific gene regulators that may have a favorable or adverse effect on the synthesis of bacterial secondary metabolites [25]. There are different criteria such as the type of media, shaking condition, incubation temperature, pH, and incubation time affecting the expression level of pyocyanin genes [7, 26]. Therefore, the production of pyocyanin was maximized and extracted at a concentration of 53 µg/ml. Conversely, the pyocyanin yield by using synthetic glucose-supplemented nutrient media was 9.3 and 5.9 μg/ml for *P. aeruginosa* R1, and *P. aeruginosa* U3, respectively [27]. According to [28], the addition of 0.2% toluene increases the pyocyanin yield up to 33 μg/ml. In the study performed by [29], pyocyanin yield using lime-cooked maize wastewater (nejayote) was 3.25 μg/ml by *P. aeruginosa* NEJ01R. The enhanced pyocyanin production of 2.56 μg/ml was achieved in King’s A medium amended with soya bean followed by 1.702 μg/ml of pyocyanin from the nutrient medium amended with sweet potato [30]. The designed modified semisynthetic (MS) media for pyocyanin yield enhancement led to increasing pyocyanin yield up to 6 μg/ml [31]. The study established by [32] showed a pyocyanin reduction by indole addition and no change was detected in the pyocyanin yield with acetate addition.

Cetrimide addition to peptone water medium led to a significant (*P* < 0.05) increase in pyocyanin production by 38.5–58.9%. In related studies, the highest proportion of *P. aeruginosa* strains producing greenish pigments was obtained using Cetrimide agar [33]. Shaking condition and incubation at 37 °C significantly (*P* < 0.05) increased pyocyanin production by (31–63.5%), and (44–68.8%), respectively. In agreement with our results, shaking conditions and incubation at 37 °C were preferred for maximum pyocyanin yield [26, 34].

The maximum pyocyanin yield, in our study, was obtained after 3 days of incubation. In agreement with studies conducted by [28, 35] which reported that 72 h was the optimal incubation period for pyocyanin production. Seeing as *P. aeruginosa* started producing other pigments that affected the production rate of pyocyanin after longer incubation time [36], these pigments include pyorubin (red brown), pyomelanin (light brown), and pyoverdine (yellow green) [37–39]. The green color of pyoverdine may be mistakenly considered an indication of pyocyanin production. However, pyocyanin is soluble in chloroform, whereas pyoverdine is not. Hence, any questionable green pigments may be confirmed by simple extraction with chloroform [40]. Therefore, chloroform extraction was conducted in our study for obtaining the most purified form of pyocyanin.

According to our study, the stability of pyocyanin conversed for one year when it stored in its lyophilized form at − 18 °C while being degraded, if it stored in its dissolved form at − 18 °C, after 60, 29, and 15 days for SDW, 0.2 N HCl, and chloroform, respectively. The degradation of pyocyanin in its dissolved form was discussed by [36] showing that pyocyanin was degraded after 2 days in chloroform, 12 days in water, and 12 days in methanol. Therefore, lyophilization (freeze-drying) was a preferred method for obtaining stable pyocyanin powder. This is due to the high stability of solid dosage forms compared to liquid formulations [41], and the difficulties associated with obtaining pyocyanin powder by chloroform evaporation [36].

The anticancer activity of pyocyanin was previously reported on different cell lines such as RD, HepG2, and PANC-1 [11, 13, 14]. Our study is the first to report the anticancer activity of pyocyanin against MCF-7 with IC_50_ equal to 15 µg/ml. Based on the criteria of the National Cancer Institute and Geran protocol in which the highly cytotoxic extracts are those with an IC_50_ ≤ 20 µg/mL [16]. Therefore, the pyocyanin extract was considered highly cytotoxic against MCF-7. The anticancer activity of pyocyanin against MCF-7 was mediated by its significant induction of apoptosis and necrosis which was confirmed by confocal scanning microscopy (Fig. 10), inverted microscopy (Fig. 12), and flow cytometry (Fig. 11). The necrotic and apoptotic activities of pyocyanin were previously observed in other studies [11, 13–15]. The apoptotic activity of pyocyanin against MCF-7 is mediated by caspase-3 activation, which promotes cell death by apoptosis [11]. According to [10, 11] pyocyanin has a low molecular weight (210 Da), it easily permeates cell membranes, where it directly receives electrons from NADH or NADPH and passes those electrons to O_2_ under aerobic conditions, resulting in the production of ROS which activates caspase-3 production.

Several studies reported the cytotoxic potential of pyocyanin, due to its ability to penetrate the biological membrane and increase ROS. Pyocyanin can enter the brain and induce neurodegeneration [42]. According to [43], pyocyanin induces metabolic disorder, liver inflammation intestinal, and barrier destruction. The oral toxicity study, in silico rodent model, classified pyocyanin in class IV (ld50 0.3–2 g/kg) [44]. In the study established by [45], pyocyanin could promote lung injury, in the *P. aeruginosa*-infected airway of cystic fibrosis patients, by decreasing the ability of α 1 Protease inhibitor (α 1PI) to control the regional activity of serine proteases. However, according to our study, pyocyanin can selectively influence MCF-7 without affecting normal cells (PBMCs), at concentrations below 45 µg/ml, as shown in (Fig. 9). Our result was supported by [46], which observed no toxicity by pyocyanin up to 50 µg/ml. The study conducted by [47] revealed that pyocyanin toxicity to mammalian cells manifested at high doses and no cytotoxicity was observed with low doses of pyocyanin [47]. The reason for pyocyanin selective cytotoxicity is that Cancer cells are more sensitive to increasing ROS compared to normal cells as cancer cells have a high basal level of ROS [48, 49].

In summary, pyocyanin production can be maximized by optimization of extrinsic factors such as the type of media, shaking condition, incubation temperature, pH, and incubation time. Purified pyocyanin may be used in treatment strategies of human breast adenocarcinoma (MCF-7), which results in decreasing the viability of cells by induction of necrosis and accelerating apoptosis via caspase-3 activation.

## Conclusion

The current investigation strongly demonstrates that pyocyanin at a concentration below 45 µg/ml could significantly inhibit the growth of human breast adenocarcinoma (MCF-7) by induction of necrosis and apoptosis. This result suggests that pyocyanin is a promising source that offers opportunities to develop a novel anticancer drug as well as further studies are required for evaluating the outcome of combing pyocyanin with anticancer drugs to assess if it can augment the action of this therapeutic category.

## Methods

### Bacterial strains

A total of 150 clinical specimens (sputum, urine, pus/wound, and blood) were provided by patients under clinical investigations in Tanta University Hospital. Collected samples were cultured on a nutrient agar medium; they were incubated overnight at 37 °C to isolate the bacteria. Pigmented colonies were selected and primarily identified by Gram staining technique and standard biochemical tests including oxidase, catalase, indole, and citrate utilization. All strains identified as *P. aeruginosa* were used for pyocyanin estimation.

### Pyocyanin determination

Pyocyanin was assessed from different *P. aeruginosa* clinical isolates as previously described by [29]. On a cetrimide agar medium, the test strains were grown overnight. In order to observe the production of pyocyanin, the optical densities of the growing cultures were then adjusted to 0.257 at OD 600, inoculated into King's A fluid medium, and incubated overnight at 37 °C in a 200 rpm orbital shaker. The cell pellets were removed by centrifugation at 10,000 rpm for 10 min, and the assay used the supernatant. Chloroform and supernatant (3 ml) were added in equal parts and vigorously vortexed. The lower chloroform layer turned blue, separated, and was then combined with the same volume of 0.2 N HCl while being vigorously vortexed. The aqueous phase was separated and the pyocyanin content was measured based on the absorbance (A) at 520 nm using a UV–Vis spectrophotometer (Genesys™ 10S, Thermo Scientific, WI, USA) by the following formula: Pyocyanin (µg/ml) = A_520_ × 17.072.

## Optimization of pyocyanin production

All the experiments in this section were performed in triplicates using 250 ml Erlenmeyer flasks containing 50 ml test medium (peptone water medium supplemented with 3% cetrimide for all experiments except the effect of media experiment), and all experimental units (EUs) were inoculated with 1 ml of inoculum culture. The effect of various factors on pyocyanin was calculated using the formulas from the previous sections. In order to determine how the media affected the synthesis of pyocyanin, King’s A fluid, nutrient broth, Luria Bertani (LB) broth, peptone water, and peptone water supplemented with 3% cetrimide were all tested. The optimal medium for pyocyanin production was then employed in the following tests. For the purpose of examining how temperature affects the formation of pyocyanin, three sets of EUs were incubated at 30 °C, 37 °C, and 40 °C. To determine the impact of agitation on the production of pyocyanin, one set of EUs was held in a static condition while the other set was shaken at 200 rpm at 37 °C. For the purpose of researching how pH affects the formation of pyocyanin, three sets of EUs were previously pH-adjusted at 7, 8, and 9 while being shaken at 37 °C. For the purpose of determining how long an incubation period affects the synthesis of pyocyanin, four sets of EUs previously pH-adjusted at 8 were incubated for 24, 48, 72, and 96 h at 37 °C while being shaken.

### Mass production and extraction of pyocyanin

The procedure previously described by [30] was modified to extract pyocyanin from *P. aeruginosa*. In brief, a previously pH-adjusted (pH = 8) peptone water medium containing 3% cetrimide was used, and incubated the sample using an incubator shaker (New Brunswick Scientific, Edison, N.J., USA) for 3 days at 37 °C. Following incubation and the generation of blue-green pigment, the broth culture was withdrawn from each of the flasks and subjected to centrifugation at 12,000 × g for 20 min at 4 °C. The clear, blue-green solution of pyocyanin was then obtained by filtering the supernatant through a 0.2-micron syringe filter. This solution was then combined with an equal amount of chloroform and vortexed for 20 secs for color change. The chloroform sinks to the bottom of the flask, and the color of the chloroform changes to blue. The blue chloroform layer was transferred to another new flask and 0.2 N HCl was added, properly mixed, and further subjected to centrifugation for five minutes at 10,000 × g. Pyocyanin transferred from the chloroform layer to the aqueous layer and its color changed to red. For facilitating the purification process as prescribed by [36], after the addition of 0.2 N NaOH drop by drop until the blue color was recovered, chloroform was then added to the aqueous solution and shaken vigorously until the blue pigment passed to the chloroform phase followed by silica gel column chromatography.

### Purification and lyophilization of pyocyanin

The extracted pyocyanin was purified using silica gel column chromatography as prescribed by [27]. The silica gel (mesh size 200–500) absorbent pigment was placed onto a column (30 cm length × 2 cm diameter) that had been equilibrated with methanol and chloroform. The pigment was eluted with a combination of methanol and chloroform. The eluted fractions were examined by scanning a UV–Vis spectrophotometer; fractions having the same *λ-max* were collected. The lyophilized form of the aqueous pyocyanin extract was prepared by adding 1% sorbitol as a cryoprotectant before freezing using the lyophilizer (SIM International, FD8-8 T, AC220-230 V, 50/60HZ, USA). The sample was frozen at − 80 °C for 12 h and then was put in the instrument with subsequent exposure to a vacuum for 24 h. The resultant pyocyanin powder was stored at − 18 °C until further use.

## Characterization of pyocyanin

According to [28] HPLC was used to characterize the pyocyanin that was generated. The sample and standard were filtered using a 0.45 µm nylon filter before 20 µl was injected. A C18 column for reverse-phase chromatography (Column Inertsil ODS-3 250 × 4.6 mm, 5 µm) was used for the chromatographic separation, with a flow rate of 0.8 ml/min and a column oven temperature of 24 °C. The mobile phase was water-adjusted pH to 2.5 by HCl: Acetonitrile (15:85%).

## Pyocyanin stability

The stability of pyocyanin was checked in Sterile distilled water (SDW), 0.2 N HCl, and Chloroform at different temperatures (25 °C in dark conditions, 4 °C, and − 18 °C) by observing the common physical properties. Pyocyanin has a blue color in alkaline and neutral pH while becomes red in color in acidic pH [50], as well as a remarkable dual solubility character of pure pyocyanin which enables it to transfer from organic to aqueous phase [6]. The concentration of pyocyanin in the three different solvents, stored in the refrigerator, was detected by days as described by [26]. The intact and degraded, after color change, pyocyanin was subjected to spectroscopic analysis over a range of 200–400 nm using a UV–Vis spectrophotometer (PG, T80 + , England) and compared to standard pyocyanin (Sigma-Aldrich, St Louis, MO, USA) as prescribed by [51]. Finally, for better estimation of pyocyanin stability, we checked the antibacterial activity against *Escherichia coli*, obtained from our lab, for the dissolved pyocyanin in SDW, day 1 lyophilized pyocyanin, the same pyocyanin solution after 60 days, after losing its blue color, stored at − 18 °C, and the dissolved pyocyanin in SDW after one year of storage its lyophilized form at − 18 °C as prescribed by [8, 52]. The resulted inhibition zones were measured and compared to each other; the experiment was repeated three times.

### Safe concentration range of pyocyanin on human peripheral blood mononuclear cells (PBMCs)

This assay was available to determine how pyocyanin affected healthy human cells [53]. Blood samples (10 ml) in sterile, heparinized tubes were taken from healthy volunteer donors in order to extract the PBMCs. Standard Ficoll-hypaque density centrifugation was used to separate peripheral blood mononuclear cells. Interface lymphocytes were collected, and two sterile PBS washes were performed on them. The MTT assay was used to determine the cytotoxic effect of pyocyanin on normal cells as prescribed by [54]. The absorbance was measured at 490 nm via a spectrophotometer (PG, T80 + , England), and the experiment was repeated three times.

### Cell Line and MTT assay

The human breast adenocarcinoma cell line (MCF-7) was utilized in this research (Sigma-Aldrich, St Louis, MO, USA), these cells were grown in a culture medium (DMEM) containing 5% fetal bovine serum, 100 UI/ml penicillin, 100 µg/ml streptomycin, and 0.2% sodium bicarbonate at 37 °C in a humidified environment with 5% CO_2_. Cells of MCF-7 were seeded in 96-well culture plates (5 × 10^5^ cells/ ml) and cultured overnight to allow for cell attachment. Cells of MCF-7 were treated with different concentrations of pyocyanin (dissolved in sterile distilled water (SDW) and always protected from light). After incubation for 24 h, MTT (20 μl) was added to each well and incubated for 4 h at 37 °C. Formazan crystals were dissolved in 150 μl of DMSO for 10 min with shaking. The absorbance was measured at 490 nm via a spectrophotometer (PG, T80 + , England), and the experiment was repeated three times [54].

### Apoptosis and necrosis detection using confocal laser scanning microscopy (CLSM)

The effect of pyocyanin on the viability of MCF-7 cells was assessed by using acridine orange AO and propidium iodide PI double-staining procedure as prescribed by [55]. In brief, cells of MCF-7 were seeded in a 12-well plate, at a density of 1 × 10^5^ cells/well, and incubated overnight, then treated with 0.5 IC_50_ and IC_50_ of pyocyanin (15 and 30 µg/ml) for 24 h. After removing the media and washing cells with PBS, the cells were stained in equal volumes (300 μL) of 100 μM AO/PI and incubated, in dark conditions, at room temperature for 15 min. Finally, stained MCF-7 cells were re-washed and examined under the fluorescence microscope (DMi8; Leica Microsystems) at × 10 magnification and the number of viable/dead cells were counted.

### Flow cytometry using annexin V-FITC/PI

Apoptosis and necrosis induction after 24 h of pyocyanin treatment (IC_50_) of MCF‐7 cells were assessed using Novocyte Flow Cytometer (Acea Biosciences, USA) as prescribed by [56]. In brief, harvested MCF-7 cells were washed and re‐suspended in PBS (100 μL), followed by staining with the solution of Annexin V‐FITC/PI (10 μl). The analysis was performed after a 20 min incubation in dark.

### Morphological study using phase contrast microscopy

The phenotypical changes of MCF-7 cells after pyocyanin treatment were detected using phase contrast microscopy. The untreated and pyocyanin-treated (IC_50_) MCF-7 cells were visualized after 24 h using an inverted microscope (Leica, Wetzlar, Germany) for the detection of the morphological changes [57].

### Real-time PCR to detect gene expression

The kit's instructions (Roche Diagnostic GmbH, Germany) were followed while extracting total RNA. Using a spectrophotometer, the concentration and purity of mRNA samples were determined. We only used samples with an OD260/OD280 ratio between 1.8 and 2.0. The used primers were Caspase-3-specific primers (forward: 5'-GTGGAACTGACGATGATATGGC-3' reverse: 5'-CGCAAAGTGACTGGATGAACC-3') and β-actin-specific primers (forward: 5'-AAGATCCTGACCGAGCGTGG-3' reverse: 5'-CAGCACTGTGTTGGCATAGAGG-3'). cDNA was manufactured using reverse transcription with a system of 20 µl. Real-time PCR reaction system: 25 µl, reaction conditions: 95 ˚C for 30 s, followed by 40 cycles of 95 ˚C for 5 s, 60 ˚C for 30 s, and 72 ˚C for 60 s. The automatic output from a real-time PCR device Rotor-Gene Q (Qiagen, USA) was used to determine the expression level of caspase-3 using β-actin as the endogenous control [58].

### Western blot analysis

Pyocyanin (IC_50_) was used to treat MCF-7 cells, and a radioimmunoprecipitation assay buffer was used to extract the total protein. Total proteins were collected, quantified, and subjected to 12% sodium dodecyl sulfate–polyacrylamide gel electrophoresis (SDS-PAGE), separated, and deposited onto a nitrocellulose membrane. After blocking the membranes with 5% bovine serum albumin (BSA), the membrane was probed with anti-active caspase-3 and anti-β-actin primary antibodies while being gently stirred overnight at 4 °C. Following three TBST buffer (Tris-buffered saline, 0.1% Tween 20) washes, the appropriate HRP-conjugated secondary antibody was added and incubated for 1 h at room temperature. A gel documentation system (Geldoc-it, UVP, England), was applied for data analysis using TotalLab analysis software (Ver.1.0.1) [16].

## Statistical analysis

The data was represented as the mean of three replicates ± standard deviation (SD). A T-test was used to compare two groups. One-way ANOVA was used to compare more than two groups and the mean comparisons were performed by Tukey’s multiple range test using GraphPad Prism version 5. Differences between means were considered significant at a p-value < 0.05.

## Data Availability

All data generated or analyzed during this study are included in this published article.
